# The Effect of Smoking and Brushing on the Color Stability and Stainability of Different CAD/CAM Restorative Materials

**DOI:** 10.3390/ma15196901

**Published:** 2022-10-05

**Authors:** Stuart Schelkopf, Caroline Dini, Thamara Beline, Alvin G. Wee, Valentim A. R. Barão, Cortino Sukotjo, Judy Chia-Chun Yuan

**Affiliations:** 1 Private Practice, University Associates in Dentistry, 222 N Lasalle St., Chicago, IL 60601, USA; 2 Department of Prosthodontics and Periodontology, Piracicaba Dental School, University of Campinas (UNICAMP), Av. Limeira, 901, Piracicaba 13414-903, São Paulo, Brazil; 3 Department of Restorative Sciences, School of Dentistry, University of Minnesota, 9-470 Moos Tower, 515 Delaware St. SE, Minneapolis, MN 55455, USA; 4 Department of Restorative Dentistry, College of Dentistry, University of Illinois Chicago, 801 S Paulina, Chicago, IL 60612, USA

**Keywords:** ceramics, CAD/CAM, cigarette, zirconia, color stability, polishing

## Abstract

This study aimed to investigate and compare the color stability and stainability of computer-aided design/computer-aided manufacturing (CAD/CAM) restorative materials in their glazed (G) and polished (P) state when exposed to cigarette smoke, as well as after brushing. Three CAD/CAM restorative materials were investigated: lithium disilicate CAD (LD), zirconia (Zr), and Telio PMMA CAD (PMMA), according to their surface finishing and assignment to cigarette smoking exposure or soaking in the saliva (control) group. The color change (∆E) was calculated before and after the intervention performed for all specimens, using the L*a*b values to quantitatively assess the shade differences. Statistical analysis was performed using one-way repeated measures ANOVA and Bonferroni multiple comparison analysis (α = 0.05). The surface finishing did not influence the materials’ stainability. Color change was noted after smoking, LD and Zr-G and Zr-P had a comparable color change (*p* > 0.05), while PMMA presented lower ∆E values (*p* < 0.05). After brushing, all specimens had a significant color change that was high for LD-G and LD-P, and Zr-G, compared with Zr-P and PMMA (*p* < 0.05). In conclusion, the exposure to cigarette smoke showed that LD, Zr, and PMMA are all susceptible to staining, but brushing decreases surface staining.

## 1. Introduction

The use of ceramics in restorative dentistry is highly popular because of their biocompatibility, pleasing aesthetic, insolubility, and hardness [1]. These ceramic restorations can be fabricated by both traditional laboratory methods and computer-aided design/computer-aided manufacturing (CAD/CAM), both being able to provide comparable mechanical and optical properties, as well as marginal adaptation [2]. The advent of CAD/CAM technology enabled materials such as silica-based ceramics, infiltration ceramics, oxide high-performance ceramics, and methacrylate-based polymers to be mass produced in ingots, leading to higher levels of homogeneity and increased consistency [3]. Moreover, high-strength materials such as zirconia, which were previously unusable due to the structural lattice conformation, can now be prefabricated and milled to create ceramic restorations [4].

There are several CAD-available materials, such as ceramics, alumina, zirconia, Telio PMMA, poly(etheretherketone) (PEEK), among others. Within the available materials, one of the most popular is CAD lithium disilicate (LD). The LD ceramic has evolved to develop today’s restorative material IPS e.max, which is both pressed and milled and uses a two-stage crystallization process for fabrication [1,5]. Another popular CAD/CAM available material often used for restorations is yttria-stabilized zirconia (Zr). Zr powders are prepared and used by the manufacturers to press and fabricate pre-sintered blanks used for milling CAD restorations [4,6]. Telio PMMA CAD (PMMA) is another material that exists as an already-polymerized poly (methyl methacrylate) material for provisional restorations. The CAD process of fabricating these restorations bypasses polymerization shrinkage, which provides improved properties for the material, including flexural strength enhancement, stain resistance, and intra-material consistency.

All these restorative materials need to meet aesthetic and functional demands for the treatment success. Previous studies evaluating CAD materials exposed to different beverages, such as coffee, tea, and red wine, have demonstrated that materials are all susceptible to staining [7,8,9,10,11]. Moreover, another common cause of extrinsic staining is cigarette smoking [12]. In 2019, 3.6% or 8.7 million U.S. adults smoked cigarettes some days or every day [13]. From this, it is evident that cigarette smoking remains highly prevalent amongst today’s population, and this habit greatly affects the long-term esthetic outcomes of restorations [14]. The color stability and stainability are critical properties; the first is related to color change over time in saliva, while stainability is the color change due to staining by a colorant. Color changes might be interpreted according to perceptibility (PT) and acceptability (AT) thresholds, which can guide the selection of dental materials, evaluate their clinical performance, and interpret visual and instrumental findings [15,16]. In dentistry, color change PT and AT were found to be ΔE = 1.2 and ΔE = 2.7, respectively [16].

In addition, functional demands, including surface finishing, adjustments, and polishing after crystallization and sintering, may lead to higher reported roughness values of CAD materials, which can also change materials’ properties, including an increase in stainability, as well as affect opposing teeth [17,18]. Clinically, it has been demonstrated that restorative materials thickness, luting agents placed, and dentist clinical experience are paramount for the treatment longevity and patient satisfaction [19,20,21]. Therefore, even with the benefits introduced by the CAD/CAM technology with higher homogeneity and structural properties of the material, it is essential to consider all these factors for the quality of the restorative treatment and to have more reliable and lasting restorations.

Previous studies have provided insight into the relative long-term color stability and stainability of CAD/CAM-available materials exposed to different beverages, post-aging periods, cigarette smoke, and smokeless tobacco [7,8,9,10,14,22,23,24]. However, the color stainability specifically of LD, Zr, and PMMA both polished and glazed state and when exposed to cigarette smoke have not been previously tested. Therefore, this in vitro study aimed to investigate and compare well-established CAD/CAM restorative materials (LD, Zr, and PMMA), regarding color stability and stainability in their polished and glazed state. In addition, the study investigated the color changes of the surfaces when exposed to cigarette smoke, as well as stain removal after brushing.

## 2. Materials and Methods

### 2.1. Preparation of the Test Specimens

For the specimen’s preparation, three CAD/CAM restorative materials were investigated in this study: LD (Shade Medium Opacity 4, IPS e.max CAD; Ivoclar Vivadent AG, Schaan, Liechtenstein), Zr (Shade Zr Translucent Pure, Wieland Zenostar Translucent; Ivoclar Vivadent AG, Schaan, Liechtenstein), and PMMA (Shade LT A3.5, Telio^®^ PMMA CAD Ivoclar Vivadent Inc., Schaan, Liechtenstein). From the raw restorative pucks and ingots, 40 LD CAD discs (ISO 6872: 2015), 40 Zr discs (ISO 6872: 2015), and 20 PMMA discs (ISO 10477:2018) were prepared using a cutter (IsoMet 1000 Precision Cutter; Buehler, Lake Bluff, IL, USA). Each disc was cut at a setting of 2 mm thickness, and the discs were cut with the saw under water cooling and at a speed of 200 rpm. The final dimensions of the specimens were: 12 × 14 × 2 mm for LD and 15 × 19 × 2 mm for PMMA, and 10 × 16 × 2 mm for Zr. Final dimensions of each material were based on the block size from the manufacturer.

**Figure 1:**
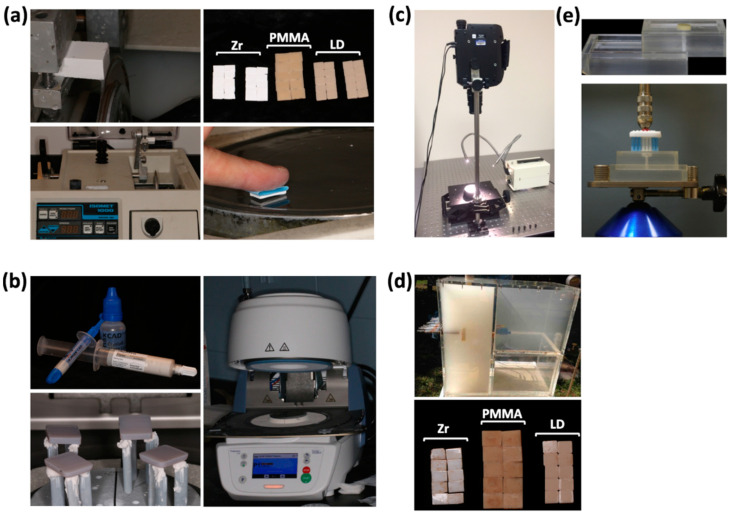
(**a**) Specimen preparation with sectioning and polishing; (**b**) specimen glazing and crystallizing; (**c**) spectroradiometer analysis; (**d**) smoking chamber used in the study and specimens after smoking exposure; (**e**) standardized sliding device for brushing and toothbrush head fixed on the paralellometer (reprinted with permission from Elsevier (License Number: 5392551271768)). Used acronyms: LD—lithium disilicate CAD, Zr—zirconia, PMMA—and Telio PMMA CAD.

After sectioning, the specimens were serially smoothed using an ECOMET Polisher/Grinder (Buehler, Lake Bluff, IL, USA) with silicon carbide grinding paper from grit P800 to P4000 under water to eliminate any surface irregularities following the previous protocol [25] (Figure 1a). The resulting thickness of the discs remained at 1.8 +/− 0.1 mm, measured with calipers. After this preparation, all specimens were steam cleaned in 5% NaOH solution for 10 min, ultrasonic cleaning for 1 h, and dried by nitrogen gas before glazing and crystallizing. All LD specimens were crystallized and glazed (IPS e.max CAD Crystall/Glaze Paste) following the manufacturer’s recommendation (850 °C, Programat CS2; Ivoclar Vivadent AG, Schaan, Liechtenstein). All Zr specimens were sintered at 1540 °C for 2 h and 16 min and again at 100 °C for 8 h and 35 min in a high-temperature sintering furnace (Dentsply Sirona, Charlotte, NC, USA) following the manufacturer’s recommendation. Characterization (IPS shade 3) and glaze (IPS e.max Ceram Glaze FLUO paste, Ivoclar Vivadent, Schaan, Liechtenstein) layers were applied, and the firing was performed at a 6-min pre-drying temperature of 403 °C with a heat rate of 45 °C/min. The vacuum start and stop temperatures were 450 and 769 °C, respectively, with a 1-min hold time. The PMMA does not require any additional processing after milling (Figure 1b). All specimens were then randomly assorted into their respective groups.

Specimens were polished to simulate the clinical adjustment and polishing of a restoration. Each specimen was lightly abraded with a fine diamond bur with an electric handpiece at 10,000 rpm under water irrigation. Subsequently, the ceramic specimens (LD and Zr) were polished with the Optrafine F, followed by Optrafine P, and finally Optrafine HP with a contra-angle handpiece at 10,000 rpm. The Optrafine F and P polishing was completed under water irrigation; however, the Optrafine HP polishing was utilized with diamond polishing paste, as instructed within Ivoclar Vivadent’s protocol. The PMMA specimens were polished with the Optrapol under water irrigation at a speed of 10,000 rpm, as instructed in their protocol (Ivoclar Vivadent).

### 2.2. Sample Testing and Color Measurement

Baseline color measurements were performed using a spectroradiometer PR 650 (Photo Research Inc., Chatsworth, CA, USA) (Figure 1c). The spectral reflectance of each sample was measured from 380 to 780 nm wavelengths at a 5 nm interval with an optical configuration of 45 degree illumination and 0 degree observer angle. The spectral data for each specimen were then recorded and converted to CIELAB values for a second observer and D65 illumination, which was also recorded. The spectroradiometer has been shown to be valid and repeatable when measuring the color of dental restorative materials [26,27].

Upon completion of initial color measurements, the assigned specimens were subjected to smoke testing by using a smoking chamber developed by the Department of Restorative Dentistry of Piracicaba Dental School (registered under Number 01810012043 INPI—National Institute of Industrial Property) and constructed according to specifications that were outlined in a previous study [24]. The experiment specimens were subjected to the following smoking conditions: the negative pressure chamber obtained through a vacuum pump simulated an inhalation condition that starts and conducts smoke through glass cannulas aiming to allow it to circulate and deposit the chemical products on the specimens [28]. Each specimen was positioned 1 cm from the glass cannula that introduced the cigarette smoke into the chamber, being in total 10 cigarettes and 10 specimens at a time. Cycles of smoking were scheduled in time intervals, replicating the typical smoking behavior of a smoker. The specimens were exposed to the smoke for 3 s per inhalation. Ambient air was then inhaled, replacing the smoke in the chamber. Specimens were subjected to the smoking of 1 pack of cigarettes (Marlboro) per day for a total of 10 days (Figure 1d). In the intervals between exposures, the specimens were stored in artificial saliva (0.4 g/mL KCl, 0.4 g/mL NaCl, 0.906 g/mL CaCl_2_·2H_2_O, 0.690 g/mL NaH_2_PO_4_·2H_2_O, 0.005 g/mL Na_2_S·9H_2_O, and 1 g/mL urea (pH = 7.0)) at 37 °C to simulate clinical conditions when they were not being subjected to the smoking conditions. Every 24 h, the specimens were washed with distilled water and resubmerged in fresh artificial saliva solution to prevent sedimentation. The control specimens (non-smoked) were stored in artificial saliva solutions with the same composition for 10 days without removal. After the 10 days of smoking exposure, color measurements were performed in the same way as the baseline color measurements, and the data were recorded.

In addition to spectroradiometric analyses, the specimens were subjected to brushing after smoke exposure to remove any gross residue and to measure the removal of chemical residues after brushing on different surfaces (Figure 1e). A constant-pressure, lateroscursive tooth brushing apparatus was constructed from specifications outlined in a previously published related study [24]. This apparatus was constructed from a dental surveyor, with the addition of an attached toothbrush head to the mandrel and an acrylic base for specimen placement on the surveying table. The lower movable table with an acrylic base presented 4 spaces for fixation of the specimens, and they were fixed at the level of the brush tips so that only the specimen’s surfaces were in contact with the tips. Brushing of the specimens included 20 forward–backward movements of the table on the surveying platform with constant pressure of the toothbrush bristles against the specimen, with water available around the specimen up to the level of the specimen surface but not submerging the specimen under water. This is to cleanse the specimen and bristles throughout the brushing between strokes. After brushing, final post-brushing color measurements were made, and the data were recorded.

The color change (∆E) was calculated before and after the intervention performed for all specimens. Change in color was calculated between baseline and post-soaking on saliva or post-smoking, as well as between post-soaking on saliva or post-smoking and post-brushing using the L*a*b values to quantitatively analyze the shade difference of each treatment group. Color differences were calculated using the ∆E formula:

$$\Delta E=\sqrt{{\left(\Delta L\right)}^{2}+{\left(\Delta a\right)}^{2}+{\left(\Delta b\right)}^{2}}$$

where ∆L = L*f − L*i, ∆a = a*f − a*i, and ∆b = b*f − b*I, and i is referred to as the initial color measurement and f as the final color measurement.

**Figure 2:**
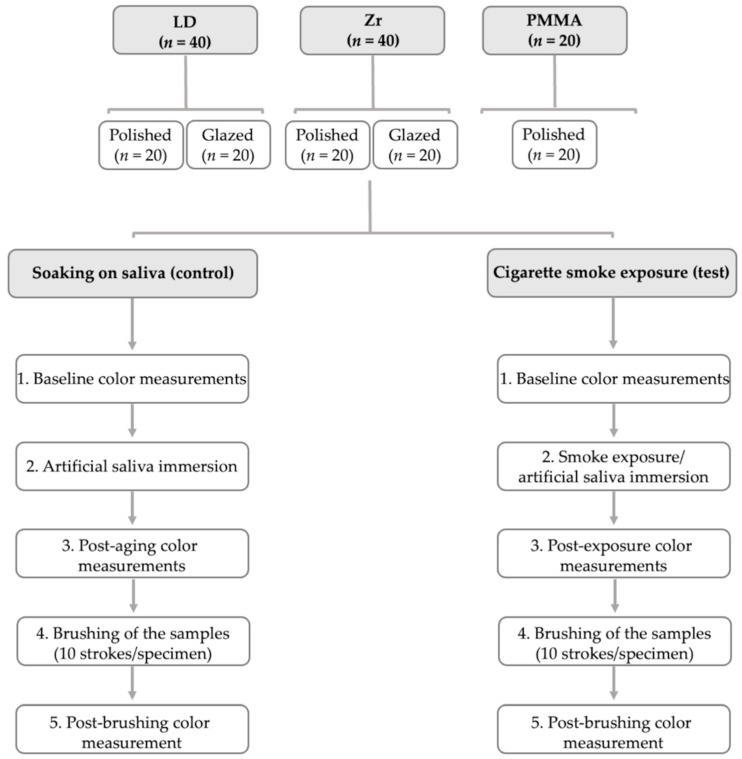
Schematic diagram of the experimental design. Lithium disilicate (LD), zirconia (Zr), and Telio PMMA (PMMA).

The schematic diagram of the experimental design is detailed in Figure 2.

### 2.3. Statistical Analysis

Statistical analyses were performed with statistical software (IBM SPSS Statistics for Windows, v. 21.0, IBM Corp., Armonk, NY, USA). The Shapiro–Wilk method was applied to test the normality of all response variables. To compare the different CAD/CAM restorative materials and to verify the influence of evaluation time (baseline, saliva or smoking, and brushing) on the response variables (L*, a*, b*, ∆E, ∆L, ∆a, and ∆b), one-way repeated measures ANOVA was performed. Multiple pairwise comparisons were examined with the Bonferroni post-hoc test. A mean difference significant at the 0.05 level was used for all tests.

## 3. Results

### 3.1. Color Measurement

**Figure 3:**
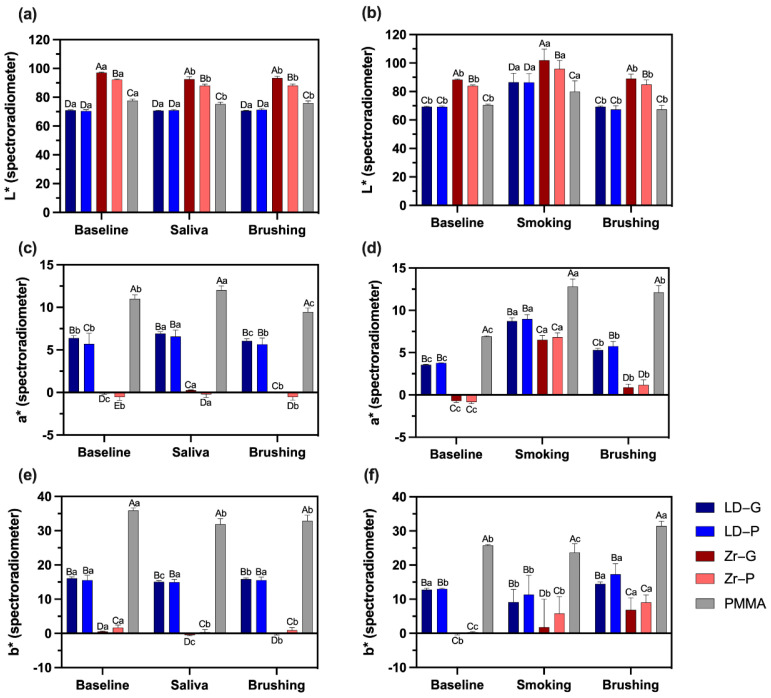
L*, a*, and b* values according to the experimental groups (*n* = 10) and timing of color measurement, graphs on the left represent stability and on the right stainability; (**a**) L* values in the baseline, after soaking on saliva, and after brushing; (**b**) L* values in the baseline, after smoking, and after brushing; (**c**) a* values in the baseline, after soaking on saliva, and after brushing; (**d**) a* values in the baseline, after smoking, and after brushing; (**e**) b* values in the baseline, after soaking on saliva, and after brushing; (**f**) b* values in the baseline, after smoking, and after brushing. Dissimilar letters indicate statistically significant differences. Capital letters compare different surfaces within the same moment of evaluation (baseline, saliva/smoking, or brushing). Lower case letters compare the same surface at different moments. Lithium disilicate glazed (LD-G), lithium disilicate polished (LD-P), zirconia glazed (Zr-G), zirconia polished (Zr-P), and Telio PMMA (PMMA).

The statistical analysis of the luminosity values (L*) showed that the different materials have different degrees of luminosity (Figure 3a). Interestingly, the Zr group showed different luminosity when glazed compared with polished (*p* < 0.001). Considering the color measurements for the baseline, and after soaking on saliva and brushing, it is observed that Zr-G and Zr-P, as well as PMMA surfaces, increased luminosity after soaking on saliva and brushing compared with the baseline analysis (*p* < 0.005) (Figure 3a). Smoking decreased the luminosity of all surfaces (*p* < 0.001); however, after brushing, the surface luminosity was comparable to the baseline values (Figure 3b).

For a* values indicating red-green color components, positive values characterizing the red component were higher for PMMA, followed by LD-G and LD-P, while Zr-G and Zr-P presented negative values characterizing the green component (Figure 3c). After soaking on saliva, the red component increased for all surfaces (*p* < 0.05) compared with baseline, but brushing reduced a* values comparable to the baseline or even to lower values for LD-G (*p* = 0.002) and PMMA (*p* < 0.001) groups (Figure 3c). Smoking considerably increased the red component for all surfaces (Figure 3d), but brushing was not completely effective in reducing a* values to the same level as the baseline prior to cigarette exposure (*p* < 0.05).

Yellowish appearance represented by b* values was higher for PMMA, followed by LD and Zr surfaces (*p* < 0.05) (Figure 3e). Soaking on saliva changed the yellowish appearance of all surfaces to a lighter shade (*p* < 0.05), except for LD-P (*p* = 0.217). Smoking did not increase the yellowish appearance of the surfaces; however, brushing after smoking induced all groups to have an increased yellow aspect (*p* < 0.05) (Figure 3f).

### 3.2. Color Change

**Figure 4:**
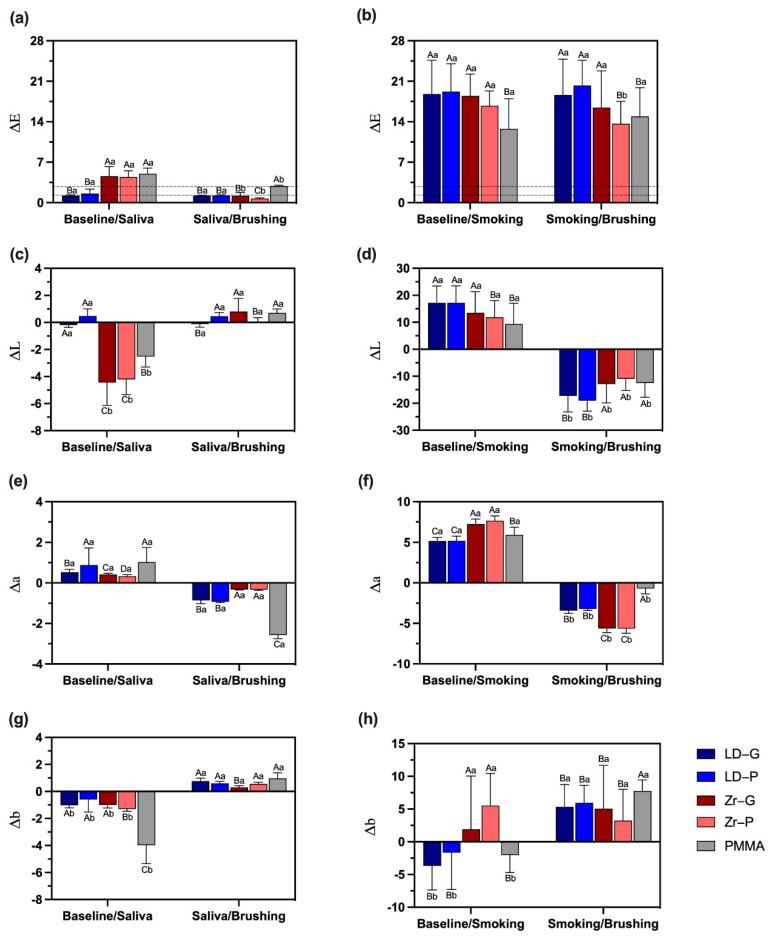
ΔE, ΔL, Δa, and Δb values according to the experimental groups (*n* = 10) and moments of color measurement; (**a**) ΔE values indicating sample color changes between soaking on saliva and baseline, lower dashed line representing the perceptibility threshold (ΔE = 1.2) and higher dashed line representing the acceptability threshold (ΔE = 2.7); (**b**) ΔE values indicating sample color changes between brushing and after smoking, lower dashed line representing the perceptibility threshold (ΔE = 1.2) and higher dashed line representing the acceptability threshold (ΔE = 2.7); (**c**) ΔL values indicating sample color changes between soaking on saliva and baseline; (**d**) ΔL values indicating sample color changes between brushing and after smoking; (**e**) Δa values indicating sample color changes between soaking on saliva and baseline; (**f**) Δa values indicating sample color changes between brushing and after smoking; (**g**) Δb values indicating sample color changes between soaking on saliva and baseline; (**h**) Δb values indicating sample color changes between brushing and after smoking. Dissimilar letters indicate statistically significant differences (one-way repeated measures ANOVA). Capital letters compare different surfaces within the same moment of evaluation (baseline, saliva/smoking, or brushing). Lower case letters compare the same surface at different moments. Lithium disilicate glazed (LD-G), lithium disilicate polished (LD-P), zirconia glazed (Zr-G), zirconia polished (Zr-P), and Telio PMMA (PMMA).

The results obtained for color change are observed in Figure 4. As regards the ΔE values, all the groups presented a change in color under different experimental conditions. For the color change between samples after soaking on saliva and baseline, Zr and PMMA surfaces exhibited greater color change compared with LD surfaces (*p* < 0.001) (Figure 4a). Specimens soaked in saliva followed by brushing had a slight color change, mean ΔE values ranged from 1.20 to 5.01. Smoking and baseline color change was high for all groups, LD-G, LD-P, Zr-G and Zr-P had a comparable color change (*p* > 0.05), while PMMA presented lower values (*p* < 0.05) (Figure 4b). After brushing, all samples had a significant color change that was high for LD-G (mean ΔE value 18.62 ± 6.20), LD-P (20.26 ± 4.37), and Zr-G (16.46 ± 6.32), compared with Zr-P (13.67 ± 3.87) and PMMA (14.91 ± 4.98) (*p* < 0.05) (Figure 4b).

As regards the analysis of ΔL, the lightness difference between samples after soaking on saliva and baseline was lighter only for LD-P (mean ΔL value 0.47 ± 0.53), whereas for the other surfaces, the negative lightness difference indicates lighter surfaces. Zr-G and Zr-P presented significant lower mean ΔL values than LD-G, LD-P, and PMMA (*p* < 0.05) (Figure 4c). Specimens soaked in saliva followed by brushing had an increase in mean ΔL values. The lightness difference between samples exposed to smoking and baseline indicate that smoking decreases the luminosity, and it was significantly higher for LD-G, LD-P, and Zr-G than for Zr-P and PMMA (*p* < 0.05) (Figure 4d). After brushing, all samples had a significant luminosity increase and it was significantly higher for LD-G, LD-P, and Zr-G than for Zr-P and PMMA (*p* < 0.05) (Figure 4d).

Concerning the redness or greenness difference (Δa), differences between samples after soaking on saliva and baseline showed that all surfaces presented redness after soaking in saliva, while samples soaked in saliva followed by brushing presented greenness. LD-P and PMMA presented significantly higher Δa values than other surfaces (*p* < 0.05), comparing differences between specimens after soaking on saliva and baseline (Figure 4e). Interestingly, PMMA soaked in saliva followed by brushing presented the greater greenness compared with other surfaces (*p* < 0.001). For specimens exposed to smoking, Zr-G and Zr-P presented significantly greater Δa values compared to other test surfaces (*p* ≤ 0.001) (Figure 4f). After brushing, Zr-G and Zr-P exhibited significantly higher greenness than other surfaces (*p* < 0.001) (Figure 4f).

For the blueness-yellowness difference (Δb), all groups showed negative differences indicating blueness after comparison between specimens after soaking on saliva and baseline (Figure 4g). PMMA was the surface with the highest blueness difference compared with other groups (*p* < 0.001). Specimens soaked in saliva followed by brushing presented slight Δb differences with comparable results (*p* > 0.05), except for Zr-G group that showed the lowest Δb difference (*p* > 0.05). After smoking, differences between smoking and baseline showed different behavior for the different surfaces (Figure 4h). The groups LD-G, LD-P, and PMMA presented negative differences, while Zr-G and Zr-P presented positive differences. The blueness-yellowness difference for samples after smoking followed by brushing indicated that all specimens had positive differences characterizing yellow surfaces. PMMA was the surface with the greater Δb compared with other surfaces (*p* < 0.05) (Figure 4h).

## 4. Discussion

Color stability and cigarette smoke stainability before and after brushing of different restorative CAD/CAM materials were investigated. According to the results, it is evident that the staining of the tested materials is a concern for clinicians. The polished finish of LD or Zr did not lead to increased stainability when compared with the glazed surface. The exposure to cigarette smoke showed that LD, Zr, and PMMA are all susceptible to staining, but different behaviors were observed in relation to the three axes of CIE L*a*b* system. In general, the exposure to cigarette smoke followed by brushing decreased the staining of all tested restorative materials.

The color measurements of different CAD/CAM restorative materials showed that after smoking, there was an increase in the L* values for all groups, indicating darker surfaces after cigarette smoke exposure. However, brushing after smoking increased the luminosity to values comparable to the baseline for all surfaces. Concerning the red-green color components, smoking increased a* values of all surfaces, but brushing reduced the redness, although it was not fully effective at reducing the redness to the same level as the baseline. For the yellowish appearance, it was not higher after cigarette smoke exposure, but smoking followed by brushing increased the yellowing of the surface. Our findings corroborate with previous studies that exposure to smoke and tobacco products can trigger surface changes in ceramic restorations [14] and dental composites [24], leading to a reduction in the luminosity and a darker color in the specimens, with increased L* values and shift into redness and darker color clinically. The pigmentation in these materials may be due to the tar and metals, such as arsenic, lead and cadmium, present in tobacco, along with dark components of smoke that are deposited on the materials surface, being responsible for the change in surfaces color and luminosity [12]. Moreover, aromatic hydrocarbons present in cigarette smoke tar can damage surfaces and increase stainability. In addition, it is also noteworthy that changes in temperature alter the staining of ceramics [29]. Concerning the higher yellowish appearance of surfaces exposed to cigarettes after brushing, it is hypothesized that the presence of smoke residues affected the color measurement, and brushing allowed a more real measurement of staining. However, because the different materials had different initial L*a*b* values, it is important to evaluate the color change in the specimens as ΔE values, rather than the final post-smoke exposure L*a*b* values.

Concerning the color changes, lightness, redness-greenness, and blueness-yellowness differences between samples exposed to smoking and baseline were different between groups. Considering the three-dimensional axes L*a*b* for the color change (ΔE), a strong color change was observed in the materials from baseline to post-saliva immersion, as well as from baseline to post-smoke exposure, with values above the PT and AT thresholds [15,16]. Zr and PMMA presented higher color change than AT from baseline to post-saliva immersion even before smoking exposure. These results may be related to possible micro structural changes occurring on the Zr and PMMA surfaces after the aging protocol with immersion in saliva [30]. However, it is not possible to confirm the presence of surface changes since no additional tests were performed to evaluate a potential superficial modification after saliva immersion. After exposure of the specimens to the cigarette smoke in the chamber, large collections of cigarette smoke residue were noted on the surfaces of the samples, leading to high reported average ΔE values between 12.8 and 19.2. LD and Zr, both glazed and polished, presented the highest ∆E values compared with PMMA after cigarette smoke exposure. The higher color changes for LD and Zr glazed and polished surfaces after smoking may be related to the different compositions and properties of the tested materials. LD composition includes 70% of crystals embedded in a glassy matrix, the internal crystal structure of LD is rather rough and heterogeneous. When fabricated, this material is subjected to milling by diamond burs to obtain a restoration, as designed via CAD. For LD, the presence of phases other than lithium disilicate, such as lithium trisilicate, might have induced additional grain boundaries, facilitating water and pigment diffusion and rendering the material prone to changes in its luminosity [23]. However, Zr has a reported internal cubic and tetragonal structural lattice that is specific to Zr material. After smoking, there was a significant color change, mainly related to lightness and redness change, compared with the samples in the baseline. It has been reported that the low-temperature degradation can lead to structural disintegration of Zr, surface roughness, and development of microcracks, which can enhance the scattering of incident light and reduce the translucency of the material [22,31]. Still, it has been reported that changes in the Zr microstructure with the aging process may be associated with the changes in light reflection of monoclinic crystals themselves and at the boundaries between monoclinic and tetragonal crystals [32]. Conversely, PMMA presented lower color changes, and it may be related to its structural properties, chemical composition, and fabrication process different from ceramics. Because PMMA is a methacrylate polymer, its fabrication process includes free-radical polymerization under high heat and pressure. PMMA CAD/CAM is processed in a controlled environment, leading to an inherent increased homogeneity and an increased degree of polymerization that can contribute to great color stability. However, considering the three-dimensional axes L*a*b* for the color change, PMMA presented higher lightness changes than other materials. This can be explained by the polymers present in the composition of PMMA that are known to uptake water and, therefore, may be more prone to absorbing the pigments of staining solutions and result in a more yellowish surface [33].

For the processing of these materials, when rendered via CAD/CAM technique, LD, Zr, and PMMA are milled and present a surface roughness characterized by the coarseness of the burs used for milling as well as the material’s structural properties. Subsequently, glaze is applied to the restoration to provide a final material finish. However, dissimilar to ceramics, PMMA is not glazed subsequently but rather polished to provide the final restoration. When evaluating the stainability of the materials, it is important to assess the stainability in relation to the finishing of the material [34]. When analyzing the polished vs. glazed LD data, surface finish, color change, and time did not show a statistically significant difference. Looking at the effect of smoking behavior, smoking statistically promoted color alteration of LD material independent of the surface finishing. Regarding the Zr specimens, the same pattern was observed with no differences in the surface finish. Studies have reported an increased surface roughness associated with polished finish compared to glazed finish [19,20,21,22,23,35,36]. The overall consensus on the correlation between roughness and stainability is inconclusive yet favors a direct relationship between surface roughness and stainability when exposed to staining agents. Some found no statistically significant interaction between roughness and stainability [37].

Based on our results, it is evident that all materials studied were susceptible to staining when exposed to cigarette smoke. This finding is consistent with previous studies investigating the stainability of ceramic restorative materials [23,24,34]. However, there are limitations to the present study. The overall accumulation of cigarette smoke residue onto the specimens’ surface is one of the limitations. Because of the nature of cigarette smoke, residue tends to accumulate in a heterogeneous manner, leading to inconsistencies within each specimen’s post-exposure resulting surface. Another limitation is related to the surface finish; upon the polishing of the glazed surfaces of the LD and Zr specimens, the resulting surface roughness and degree of glaze removal may affect the final surface character of the specimens. In addition, only one manufacturer’s materials were included in our study. As other materials have different physical and mechanical properties, the effect of smoking and brushing on the color stability and stainability may be different. Because the specimens were polished with rubber burs rather than adjusted with coarse diamond burs, the glaze was likely minimally modified. Another limitation of this study involves the study environment conducted. Since this is an in vitro study, the environment in which the smoking and soaking occurred was carefully constructed to best replicate the oral environment; however, the oral environment is impossible to replicate exactly in an in vitro study. Therefore, careful speculation and hypotheses of these materials’ performance in an oral environment should be considered.

Finally, this study brings new insights into the stainability of different CAD/CAM restorative materials after cigarette smoke exposure, as well as the removal of excess residue after brushing the different materials. Therefore, the present in vitro study can provide guidance to clinicians in the choice of appropriate CAD/CAM restorative materials for smoker patients for greater longevity and better esthetic outcomes. However, with the constant development of new CAD/CAM available materials, additional research on color stability and stainability relating to newer materials is warranted. Other staining exposures, such as coffee, wine, and certain mouth rinses, would provide a more comprehensive, multi-factorial insight into the color stability and stainability of these materials as well. Correlation between the color stability and stainability to surface roughness should also be considered.

## 5. Conclusions

Although the processing of the CAD/CAM available materials has improved its homogeneity, staining is still an issue that leads to esthetic failure of our restorations over time. From the results of this study, we conclude the following:

I. The exposure to cigarette smoking of lithium disilicate, zirconia, and Telio PMMA caused a general perceivable staining of the specimens.
II. Routine oral hygiene can reduce surface stains effectively.
III. The surface finishing of lithium disilicate and zirconia, both glazed and polished, did not influence the surface stainability.

## Data Availability

The data will be available upon request.
